# Advances in the role of HCV nonstructural protein 5a (NS5A) of 3a genotype in inducing insulin resistance by possible phosphorylation of AKT/PKB

**DOI:** 10.1038/s41598-019-42602-2

**Published:** 2019-04-16

**Authors:** Faiza Shams, Shazia Rafique, Sadia Zahid, Mobeen Munir, Muhammad Idrees, Muhammad Ilyas, Tayyab Husnain

**Affiliations:** 1 Centre of Excellence in Molecular Biology, University of Punjab, Lahore, 54000 Lahore, Pakistan; 2 Department of Mathematics, Division of Science and Technology, University of Education, Lahore, 54000 Pakistan; 3 Hazara University Peshawar, Pakistan, and Centre of Excellence in Molecular Biology, University of Punjab, Lahore, 54000 Pakistan; 4 Centre for Omic Sciences, Islamia College University Peshawar, Peshawar, Pakistan

## Abstract

HCV genes interfere with host cellular genes and play crucial role in pathogenesis. The mechanism under which HCV genes induce insulin resistance is not much clear. This study is aimed to examine the role of HCV NS5A in inducing insulin resistance by examining its affect in the phosphorylation level of AKT/PKB. In the present study, HepG2 cells were transfected with HCV NS5A and after 24 hours of transfection, protein was extracted from cells that were pre induced with insulin at three different time intervals i.e. 1hour, 2 hours and 3hours. Dot Blot analysis was performed to study the phosphorylation level of AKT. Results showed that there is clear upregulation of serine 473 phosphorylation level of AKT in NS5A transfected cells as compared with control (without NS5A). In conclusion, upregulation of serine 473 phosphorylation by NS5A of HCV genotype 3a suggests that this gene impairs the normal insulin AKT/PKB signaling pathway that leads towards insulin resistance and Type 2 diabetes mellitus. Therefore, HCV non-structural protein NS5A should be considered as promising candidate to be studied in detail for HCV induced insulin resistance and should be regarded as a therapeutically important target for the prevention of chronic liver diseases.

## Introduction

Hepatitis C virus (HCV) is the leading cause of liver diseases affecting over 200 million people globally causes acute and chronic liver infection. HCV infection is accompanied with a number of pathophysiological disorders that lead to inflammation, oxidative stress, insulin resistance, endoplasmic reticulum (ER) stress, steatosis (infiltration of liver cells with fat), altered gene expression, apoptosis, fibrosis and ultimately leads to hepatocellular carcinoma (HCC)^1^. Insulin resistance thought to be major pathogenic factor of the metabolic syndrome and abnormalities such as type 2 diabetes mellitus (T2DM), obesity and dyslipidemia that are connected with high cardiovascular risk. HCV induces insulin resistance by many mechanisms. It is proposed that defects in insulin binding, IRS, AKT protein and other intracellular signaling or GLUT-4 are the possible mechanism of HCV induced Insulin resistance^2^. HCV infected patients are 25% more susceptible to develop insulin resistance than patients with other liver associated infections^3^. In HCV induced insulin resistance, there are high levels of TNF α, TGF β, IL-1, resistin and leptin but low level of adiponectin^4^.

Insulin signaling pathway is activated when tyrosine residues of IRS-1 are phosphoryalted that activates phosphatidylinositol 3-kinase (PI3K) which makes conversion of phosphatidylinositol 4, 5-bisphosphate (PIP2) into phosphatidylinositol-3,4,5-triphosphate (PIP3). Then subsequently Akt/PKB (protein kinase B), gets serine/threonine phosphorylation at the residues of Serine473 and Threonine308. This leads to translocation of glucose transporter-4 (GLUT-4) to plasma membrane and increases glucose uptake. GLUT-4 is a major isoform that controls the secretion of insulin into the bloodstream and enhances glucose uptake into muscle and adipose tissues. Any impairment or defect in activation of Akt/PKB or GSK3 causes inhibition of glucose uptake or glycogen synthesis, respectively^5^. HCV induced insulin resistance mechanisms such as production of oxidative stress, high level of proteasome activator 28 g (PA28g) and SOCS (the molecule suppressor of cytokine), down regulation of PPAR and activation of the mTOR pathways all are genotypic specific^6^. Degradation of IRS-1 occurs through ubiquitinylation of SOCS-3 in genotype 1 while in HCV genotype 3 down regulation of IRS-1 occurs by the enhancement of expression of SOCS-7^7^.

HCV proteins both structural and non-structural play crucial role in the development of insulin resistance. Previous research data explained crucial role of core protein by disrupting insulin signaling pathway through high expression of SOCS3^8^ or up regulation of IRS-1 serine phosphorylation by c-Jun N-terminal kinase (JNK) activation^9^. This results in disruption of downstream signaling and degradation of IRS-1 function. However, the role of HCV proteins other than core protein needed to be investigated. Among them, E2 is mainly responsible for viral entry and plays an important role for virus infection but the involvement of E2 in type 2 DM has not been studied well^10–12^. Nuclear factor-kB (NF-kB) and protein phosphatase2A (PP2A) molecules might be involved in activation of HCV-induced insulin resistance. HCV non-structural proteins NS3 and NS5 interact with endoplasmic reticulum (ER) that elevate Nox2 activity and cause high level of reactive oxygen species (ROS) and nitrosylated proteins. NS5A and NS5B proteins trigger toll-like receptor-4 and NFΚB pathway that stimulate production of IL-6 and TNF^13^.

NS5A plays very significant role in HCV induced insulin resistance. Previous studies proposed that HCV NS5A also produce mitochondrial ROS, stimulating NF-kB thus increasing inflammatory cytokines IL-6, IL-8 and TNF α. Many other mechanisms about NS5A role in inducing insulin resistance are mentioned but most of the previous studies are conducted on HCV genotype 1a and 2b but little work on genotype 3a. HCV associated diabetes is an increasingly serious social, economic and medical threat faced by any developing country like Pakistan. There is an urgent need to analyze the critical role of HCV protein in developing insulin resistance and HCV induced type 2 Diabetes mellitus (T2DM)^14^.

## Material and Methods

### PCR amplification

NS5ApcDNA 3.1 clone was provided by the courtesy of Dr. Sabeen sabri. It was further confirmed through PCR with gene specific NS5A forward 5′ AGCGACGATTGGCTACGTAC 3′ and NS5A reverse primer 5′AGCAGACCACGCTCTGCTC 3′using isolated plasmid DNA as template. PCR amplification was done by using 1 µl of plasmid DNA as template, 1 µL each of forward and reverse primer, 1 µL dNTPs, 5 units Taq DNA Polymerase (thermoscientific), 2.4 uL of 25 mM MgCl_2_ and nuclease free water to make up the final volume (20 µL). The cyclic profile was; Initial denaturation step at 94 °C for 5 minutes, 35 cycles of 45 seconds at 94 °C, 45 seconds for annealing at 58 °C, 1.5 minutes at 72 °C, and a final extension at 72 °C for 10 minutes.

### Transient transfection assay with pcDNA 3.1/HCV NS5A (Genotype 3a) construct

Hep G2 cells were cultured in 6 well culture plate until they attained 70% confluency. Then cells were transiently transfected with pcDNA 3.1/HCV NS5A by using transfection reagent lipofectamine 2000 (thermo scientific, CA) according to standard protocol. Cells were harvested after 24–36 hours to analyze best outcome of gene transcription and expression profile. Transfection assay was performed in triplicates.

### Insulin induction

After 24 hours of transfection, washing of cultured cells was done with 1X PBS to eliminate the cell debris followed by treatment with 100 nM Humulin insulin for time duration of 1 hour, 2 hours and 3 hours before harvesting the cells.

### Immunofluorescence

Immunofluorescence assay was performed using gene specific antibodies according to standard protocol for further validation of transient transfection cell lines.

### RNA extraction and cDNA synthesis

After 24 hours of transfection, Trizol Reagent (Invitrogen) was used for extraction of cellular RNA according to manufacturer’s atandard method. Reverse transcription was done with 1uL oligo-dt primer, 1ug of cellular RNA and added nuclease free water to make volume upto 12uL. Incubated the mixture at 65 °C for 5 min in thermal cycler and then incubated on ice for 1 min. After that 2 µL of 10 mM dNTPs, 4 µL of 5X MMulv Buffer, 1 µL of molony murine leukemia virus reverse transcriptase enzyme (Thermo scientific), 1 µL of 0.1 mM DTT were added to make volume upto 20 uL. The reverse transcription-PCR was run at 37 °C for 50 min and at 70 °C for 15 min, then stored at −20 °C.

### Expression analysis of AKT by real time PCR

Primers used for AKT amplification are forward primer:

5′ CATCACACCACCTGACCAAG 3′ and reverse primer:

5′ CTCAAATGCACCCGAGAAAT 3′. Primers used for internal control GAPDH are forward primer: 5′ ACCCAGAAGACTGTGGATGG 3′ and reverse primer:

5′ AGGGGTCTACATGGCAACTG 3′. Real Time PCR was done by using SYBR green master mix 2X (Thermoscientific) 12.5 µL, template cDNA 50 ng/µL, respective forward and reverse primer 0.75 µL each and RNase free water to make volume upto 25 µL. Cyclic profile was; Initial step at 94 °C for 5 minutes, 40 cycles of 30 seconds denaturation at 94 °C, 30 seconds for annealing at 60 °C, 30 seconds at 72 °C. Real time PCR data was analyzed using excel from three individual trials. Real Time PCR analysis was done by comparative CT method for the calculation of relative Quantification of target genes. GAPDH was taken as an internal control and the process was done in triplicates.

### Statistical analysis

Graph Pad Prism version 5.0 was used to analyze real time PCR data and 2-tail error bars represent Standard error of mean (SEM) of the data from three individual tests. Differences between means of readings were compared using Student t test. P value of <0.01 was regarded as statistically significant and represented as *.

### Protein extraction and SDS-gel electrophoresis

For protein quantification and further use in Dot Blot method, the cultured cells considered good for further processing after having 70% confluency followed by harvesting. After harvesting, the cells were then subjected to lysis by adding cell lysis buffer (70–100 µl) containing PMSF and Protease Inhibitor. Cell lysis was done by vigorous pipetting and after that placed on ice for 15 minutes followed by centrifugation at 13000 rpm for 30 min at 4 °C. Supernatant was obtained, mixed with 5X protein loading buffer and 20X reducing dye (Fermantas) after calculating extracted protein volume. Reaction tubes containing mixture boiled in water for 4–5 mints and placed on ice for 3–4 minutes. After that protein samples were loaded into 12% SDS-PAGE gel along with 8 µl protein marker (thermoscientific) and gel was run at 60 V for 40 minutes.

### Dot Blot method

After treatment of 3 hr, 2 hr and 1 hr, 1ul of protein sample and control was loaded on nitrocellulose membrane. The blot then was placed in blocking solution (5% skimmed milk) for 1 hour and washed thrice with 1X PBST. After blocking with skimmed milk, it was incubated with primary antibody overnight at 4 °C. Blot was washed three times with 1X PBST and incubated with AP/HRP conjugated Secondary antibody for 45 min and followed by three washings with 1X PBST. Then blot incubated with substrate NBT-BCIP tablet (AP conjugated secondary antibody) for 15 minutes. In case of HRP conjugated secondary antibody, it was developed with DAB tablet (1 DAB tablet and 0.1% H_2_O_2_ in 10 ml dis. Water). Six different antibodies NS5A, HPR-conjugated sec.AB, AKT, actin, AP-conjugated sec.AB, FITC (Santa Cruze) were used and actin was taken as internal control.

## Results

### Confirmation of pcDNA3.1/NS5a HCV construct

**Figure 1:**
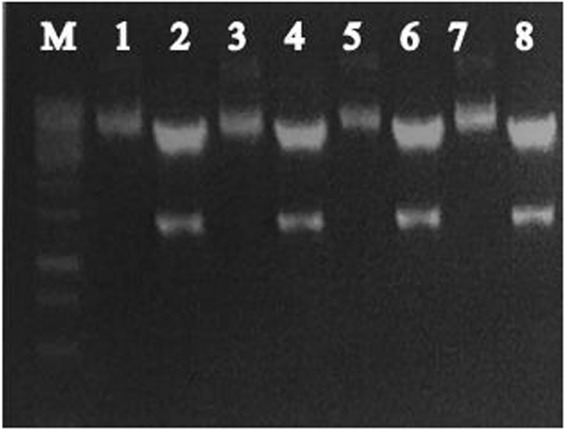
Confirmation of pcDNA 3.1/NS5A HCV construct by Restriction digestion. The pcDNA 3.1/NS5A HCV construct was double digested with *BamHI and NotI*which gives the exact product of 1356 bp and 5.4 kb and analyzed on 1% agarose gel. Lane M: 1 kb DNA ladder; Lane 1,3, 5, 7: undigested pcDNA 3.1/NS5A HCV construct; lane 2, 4, 6, 8: double digested NS5A product of 1356 bp.

The pcDNA 3.1/NS5A HCV construct was confirmed through restriction digestion by using *BamHI and NotI*which restriction enzymes that gave the exact product of 1356 bp and 5.4 kb as shown in Fig. 1.

### PCR with gene specific and vector specific primers

**Figure 2:**
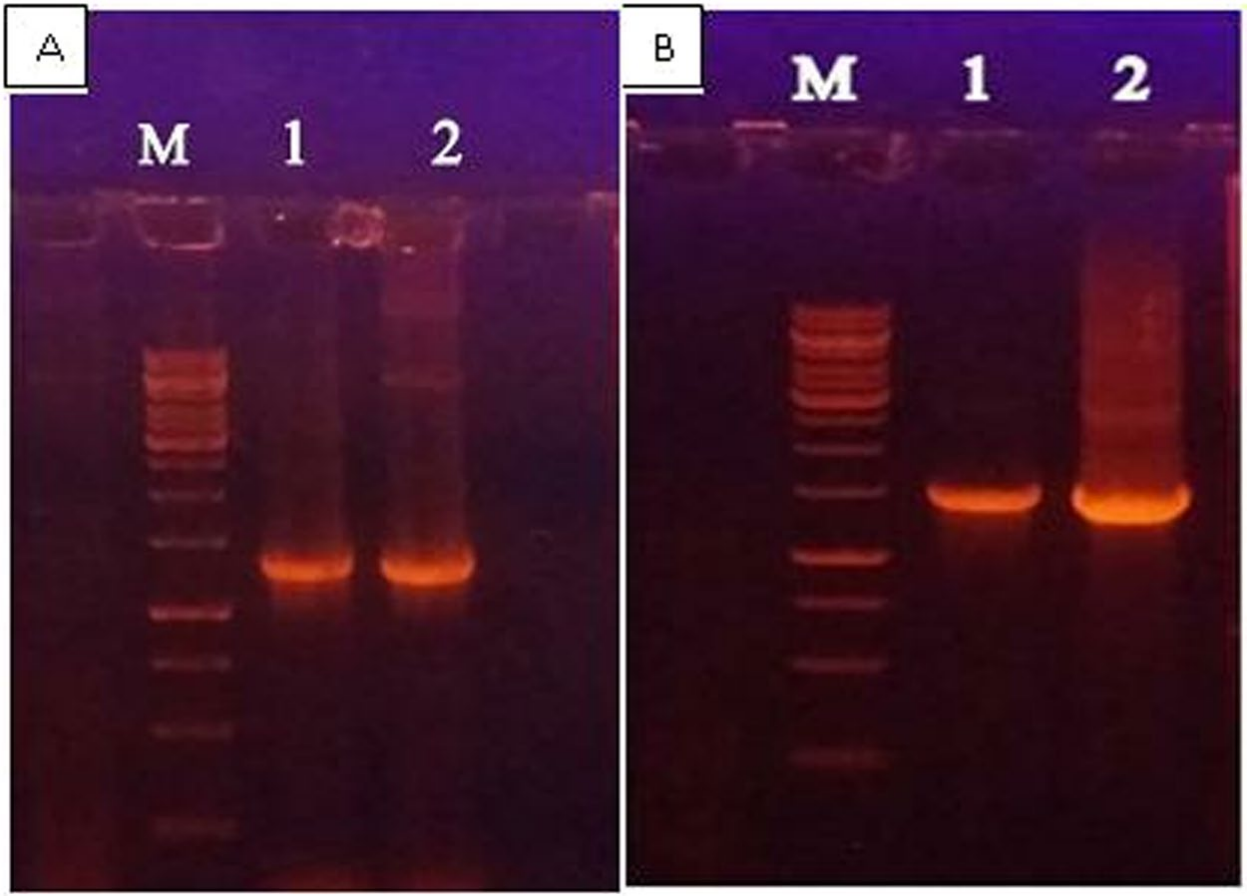
(**A**) Confirmation of pcDNA 3.1/NS5A HCV construct by PCR amplification using gene specific primers of NS5A of HCV 3a on 1.2% agarose gel. Lane M: 1 kb DNA ladder, Lane 1–2: NS5A gene of 1356 bp. (**B**) Confirmation of pcDNA 3.1/NS5A HCV construct by PCR amplification of NS5A genes using vector specific primers (T7 and BGH) on 1.2% agarose gel. Lane M: 1 kb DNA Marker, Lane 1–2: NS5Aof ~1356 Kb.

Full length NS5A gene was amplified by PCR using pcDNA 3.1/NS5A HCV as template. Gene and vector specific primers of NS5A for the confirmation of construct were used. The amplified product was analyzed on agarose gel (1.2%). Figure 2A showed the confirmation of pcDNA 3.1/NS5A HCV by successful amplification of NS5A gene i.e. 1356 bp of HCV genotype 3a. The confirmation of pcDNA 3.1/NS5A HCV by successful amplification of NS5A gene using vector specific primers is depicted by Fig. 2B.

### Sub-cellular Localization pattern of NS5A proteins

**Figure 3:**
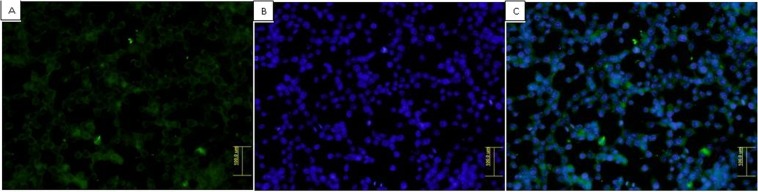
Immunofluoresence assay. (**A**) Staining with FITC. (**B**) Counter staining with DAPI&. (**C**) Merged. Transfected HepG2 cells were grown on cover slip at 37 °C. After 24 hours cells were fixed with 4% ice cold PFA, followed by blocking with 5% BSA and then incubation with primary antibody against HCV NS5A. Secondary antibody conjugated with FITC was used and performed microscopy. The results showed that NS5A protein was localized in the cytoplasm.

Immunoflorescence assay was performed to direct visualize the expression of NS5A protein of HCV genotype 3a in HepG2 cells containing the HCV sub genomic replicons. For this cells were treated with gene specific (HCV NS5A) antibodies. To check the viability of transfected cells, counter staining with DAP-I was performed and observed under microscope. Results showed that NS5A protein transiently expressed by HepG2 cells was found in cytoplasm of transfected HEPG 2 cells (Fig. 3A–C). These results confirmed that cells were alive and growing.

### Confirmation of NS5A transfection

**Figure 4:**
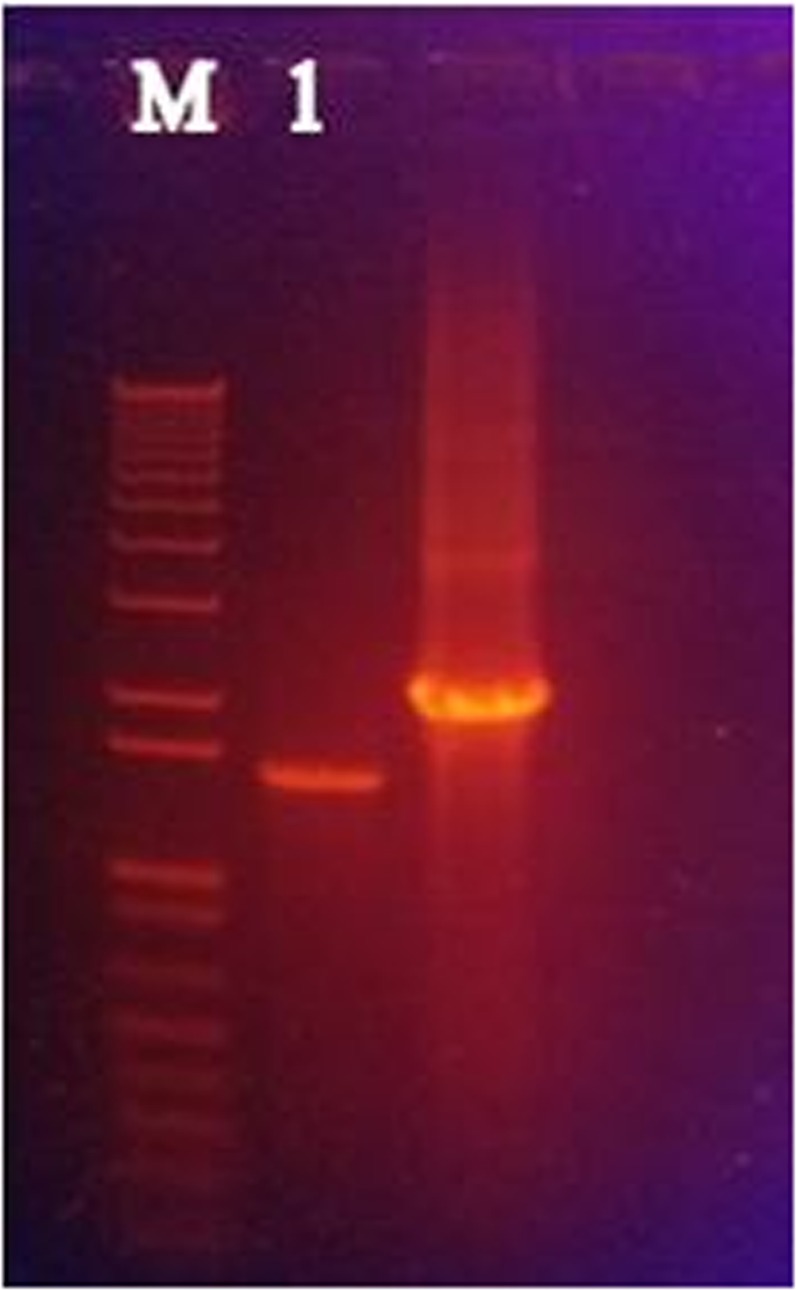
Confirmation of transfection by PCR amplification using gene specific primers. Lane M: 1Kb plus DNA Marker; Lane 1: NS5A of ~1356 bp.

After 24 hours transfection of HepG2 cells, extraction of RNA was done by using Trizol reagent (Invitrogen). Obtained RNA samples were quantified by using Nanodrop. The best quality RNA samples were used to synthesize complementary DNA (cDNA). PCR amplification was done with gene specific primers. Figure 4 depicted the precise amplification of NS5A gene of 1356 bp.

### Real time PCR

**Figure 5:**
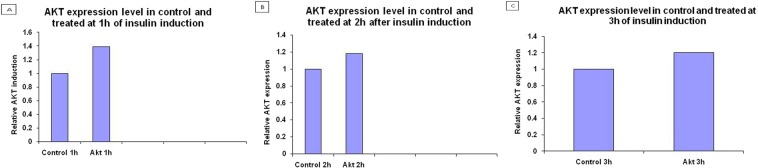
Expression analysis of Akt/PKB gene after 1,2 and 3 hours in figure (**A**–**C**) respectively of insulin induction in transfected vs. control through Real Time PCR. HepG2 cells were transfected with pcDNA3.1/NS5A HCV construct and allowed to proliferate. Insulin induction was given for 1 hour, 2 hours and 3 hours shown in figure (**A**–**C**) respectively followed by total cellular RNA extraction and cDNA synthesis after 24hrs post transfection and relative RNA determination was carried out using Real Time PCR. GAPDH was used as internal control.

After confirmation of transfection, mRNA level of AKT was checked by real time PCR at three different time intervals i.e. 1 h, 2 h, 3 h of insulin induction and GAPDH was used as an internal control (Fig. 5A–C).

### Protein Expression Profiling of NS5A transfected HepG-2 cell line and control by SDS-PAGE

**Figure 6:**
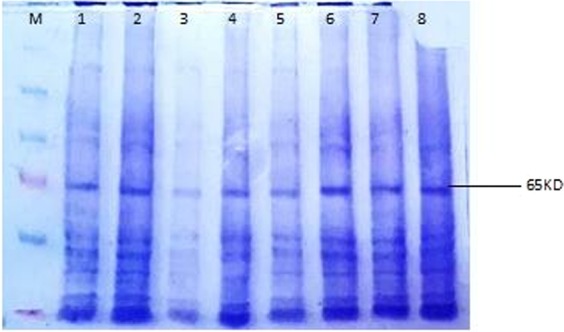
Protein Expression Profiling of NS5A transfected HepG-2 cell line and control by SDS-PAGE (12%) for Detection of Akt/PKB. The expected band of 65 KDa was observed in each of the transfected and control protein sample on the gel. Lane M: Prestained protein marker; Lane 1: Control 1 hour; Lane 2:NS5A transfected 1 hour; Lane 3: Control 2 hour; Lane 4: NS5A transfected 2 hour; Lane 5: Control 3 hours; Lane 6:NS5A transfected 3 hour.

Total cellular protein was extracted from transfected Hep G2 cells at three time intervals that is 1 h, 2 h, 3 h of insulin induction (100 nM). Protein quantification was done by Bradford assay. SDS-PAGE gel (12%) was prepared and equal amount (40 µg) of protein was loaded in each well and run at 80 V for 2 hours. To visualize the protein bands on gel, coomassie brilliant blue stain was used followed by destaining using coomassie destain solution. The expected band of AKT (65–80 KDa) was observed in each protein sample i.e. transfected and control (Fig. 6).

### DOT BLOT analysis

**Figure 7:**
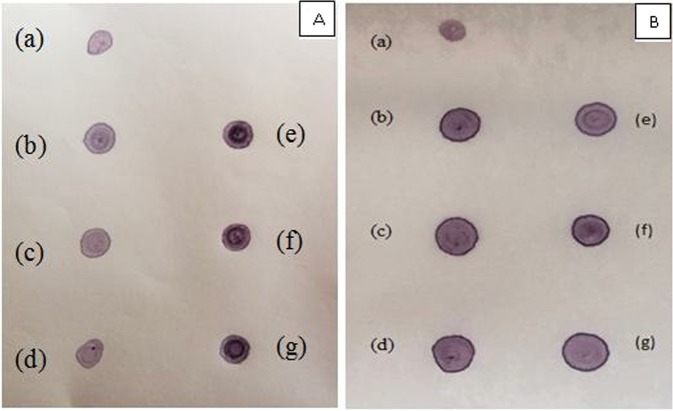
(**A**) Upregulation of serine 473 of Akt by NS5A gene of HCV genotype 3a at three different time intervals 1 h, 2, 3 h in control vs NS5A transfected. a: -ve control; b: control 3 h; c: control 2 h; d control 1 h; e: NS5A transfected 3 h; f: NS5A transfected 2 h; g: NS5A transfected 1 h. (**B**) Actin (Internal control) was confirmed in all control and transfected samples by dot bot analysis using specific antibody. a: -ve control; b: control 3 h; c: control 2 h; d: control 1 h; e: NS5A transfected 3 h; f: NS5A transfected 2 h; g: NS5A transfected 1 h.

After the observation of expected band of AKT in SDS-PAGE, protein samples subjected to dot blot assay to study the Ser^473^ phosphorylation level of AKT in the absence and presence of HCV NS5A protein. Protein samples (2 µg) were loaded on nitrocellulose membrane and blocked with BSA followed by treatment with primary and secondary antibody. The blot developed using NBT/BCIP (Sigma). Transfection of NS5A confirmed through Dot Blot by using NS5A specific antibody. In each of the transfected NS5A protein sample, the Ser^473^ phosphorylation of AKT was significantly upregulated at all of the investigated time intervals; 3 hours, 2 hours and 1 hour as shown in Fig. 7A as compared to control. Actin was used as an internal control (Fig. 7B).

## Discussion

Insulin resistance arises due to defect in metabolic processes therefore, we figured out the probable role of NS5A protein in inducing insulin resistance. To resolve the role of HCV NS5A genotype 3a in developing insulin resistance by interfering with insulin signaling pathway, AKT/PKB protein was selected as a target. To study the role of HCV NS5A genotype 3a in insulin resistance, HepG2 cell line was selected because it is not only susceptible to HCV infection but also supports its replication and the expression of HCV proteins *in vitro*^15^.

The mammalian expression vector pcDNA3.1/NS5A construct harboring full length NS5A gene was transfected into HepG-2 cell line. It has been reported that the formation of exact size of RNA confirms the functionality of the clone^16–18^. To check the expression of HCV RNA *in vitro* in HepG-2 cell line, transient transfection assay was performed using pcDNA3.1/NS5A HCV 3a construct, followed by extraction of total RNA from HepG-2 transfected cell line having the expression of NS5A gene. The extracted RNA was used for reverse transcription and cDNA was formed using gene specific primers. Observed amplified PCR product was of expected size i.e. 1356 bp for NS5A gene of HCV 3a genotype.

To study the cell localization of NS5A gene of HCV genotype 3a the immunostaining analysis of transfected cells performed. Results of immunostaining showed that NS5A protein found in cytoplasm around the nucleus. This immunostaining result also confirms the previous studies in which cell cultured and *in vivo* expression of NS5A was seen in cytoplasm of HCV transfected cells^19–21^. The immunostaining results are noteworthy as they give an account on the pattern of localization and detection of our target proteins in HCV infected cells making the clinical management and diagnosis of this deadly disease more precise as well as reflecting light on the molecular mechanism involved in pathogenesis of this virus. Real time PCR was used to determine mRNA level of AKT in NS5A transfected HepG-2 cells at three different time intervals i.e. 1 hour, 2 hours, 3 hours of insulin induction as compared with the control. With HepG-2 cell line mRNA level of AKT was not significantly altered as compared with the control. This indicates that the AKT expression in cell is parallel in transfected HepG-2 cells vs. control cells reflecting similar transcriptional and translational level.

To check the protein expression of transiently transfected Hepg2 cell lines harboring NS5A gene of genotype 3a, the crude protein was extracted after the insulin induction at three time intervals i.e. 1 hour, 2 hours and 3 hours. SDS-PAGE analysis was performed which revealed that reproducible protein expression of Hepg2 cell line transfected with NS5A of HCV after insulin induction. The expected band of 65–80 KDa was observed in each of the protein sample on the gel. This data shows that AKT is efficiently and valuably expressed in NS5A transfected HepG2 cell line. Akt/PKB-the key downstream biomolecule plays important role in multiple cell regulatory mechanisms like cell survival, metabolism, homeostasis, angiogenesis and cell proliferation^22–24^. In AKT/PKB signaling pathway, activation of AKT/PKB plays a crucial role in glucose uptake. Akt/protein, kinase B (PKB) takes the instruction from insulin receptor in the plasma membrane and passes it to the metabolic machinery inside the cell^25^. Its activated form serves as an integral component of downstream pathways of many classes of cell receptors i.e. cytokine receptors, integrins and tyrosine kinases^26^. It is suggested from previous studies that changes in the functional activity of Akt may lead to pathological conditions like oncogenesis and T2DM^23^.

Phosphorylation (Ser473) level of AKT was checked by Dot blot analysis. Significant upregulation of Serine 473 phosphorylation of AKT was observed that clearly depicts that NS5A upregulates Serine 473 phosphorylation of AKT thus impairing insulin signaling pathway. Phosphorylation Inhibition of AKT at Thr308, rather than Ser473 is thought to be culprit for impaired insulin signaling pathway thus inhibiting glucose uptake^27,28^. Consequently, NS5A of HCV 3a is a crucial protein involved in impairment of insulin signaling pathway and can hampers different metabolic pathways in cell.

## Conclusion

HCV NS5A genotype 3a contributes to insulin resistance by interfering with the functional state of AKT/PKB of insulin signaling pathway. The upregulation at serine 473 phosphorylation by NS5A HCV genotype 3a suggests that this gene impairs the normal signaling of insulin thus leading towards insulin resistance and Type 2 diabetes mellitus. Furthermore, we perceive a mechanism by which NS5A of HCV genotype 3a induces insulin resistance by significantly upregulating the critical phosphorylation at Ser473 of AKT. Therefore, HCV non-structural protein NS5A should be considered as promising candidate to be studied in detail for HCV induced insulin resistance and should be regarded as a therapeutically important target for the prevention of chronic liver diseases.

## References

[1] Jeevitha E, Sree KS, Kumar P, Kuchana V, Reddy T (2015). Hepatitis C virus infection and treatment: a mini review. *Int J Recent*. Sci Res.

[2] Eslam M, Khattab MA, Harrison SA (2011). Insulin resistance and hepatitis C: an evolving story. Gut, gut..

[3] Bugianesi E, McCullough AJ, Marchesini G (2005). Insulin resistance: a metabolic pathway to chronic liver disease. Hepatology.

[4] Sheikh MY, Choi J, Qadri I, Friedman JE, Sanyal AJ (2008). Hepatitis C virus infection: molecular pathways to metabolic syndrome. Hepatology.

[5] Hsieh M-J (2012). Hepatitis C virus E2 protein involve in insulin resistance through an impairment of Akt/PKB and GSK3β signaling in hepatocytes. BMC gastroenterology.

[6] Pazienza V (2007). The hepatitis C virus core protein of genotypes 3a and 1b downregulates insulin receptor substrate 1 through genotype‐specific mechanisms. Hepatology.

[7] Safi, S. Z., Shah, H., Yan, G. O. S. & Qvist, R. Insulin resistance provides the connection between hepatitis C virus and diabetes. *Hepatitis monthly* **15** (2014). 10.5812/hepatmon.23941 PMC4344647 25741369

[8] Kawaguchi T (2004). Hepatitis C virus down-regulates insulin receptor substrates 1 and 2 through up-regulation of suppressor of cytokine signaling 3. The American journal of pathology.

[9] Banerjee S (2008). Hepatitis C virus core protein upregulates serine phosphorylation of insulin receptor substrate-1 and impairs the downstream akt/protein kinase B signaling pathway for insulin resistance. Journal of virology.

[10] Brazzoli M (2008). CD81 is a central regulator of cellular events required for hepatitis C virus infection of human hepatocytes. Journal of virology.

[11] Mazzocca A (2005). Binding of hepatitis C virus envelope protein E2 to CD81 up-regulates matrix metalloproteinase-2 in human hepatic stellate cells. Journal of Biological Chemistry.

[12] Zhao L-J (2005). Hepatitis C virus E2 protein promotes human hepatoma cell proliferation through the MAPK/ERK signaling pathway via cellular receptors. Experimental cell research.

[13] Parvaiz F (2015). Hepatitis C virus NS5A promotes insulin resistance through IRS-1 serine phosphorylation and increased gluconeogenesis. World Journal of Gastroenterology.

[14] Bureau C (2001). Nonstructural 3 protein of hepatitis C virus triggers an oxidative burst in human monocytes via activation of NADPH oxidase. Journal of Biological Chemistry.

[15] el-Awady MK (2006). HepG2 cells support viral replication and gene expression of hepatitis C virus genotype 4 *in vitro*. World J Gastroenterol.

[16] Blight KJ, Kolykhalov AA, Rice CM (2000). Efficient initiation of HCV RNA replication in cell culture. Science.

[17] Lohmann V (1999). Replication of subgenomic hepatitis C virus RNAs in a hepatoma cell line. Science.

[18] Minakuchi C, Nakagawa Y, Kiuchi M, Tomita S, Kamimura M (2002). Molecular cloning, expression analysis and functional confirmation of two ecdysone receptor isoforms from the rice stem borer Chilo suppressalis. Insect biochemistry and molecular biology.

[19] Kasprzak A, Adamek A (2008). Role of hepatitis C virus proteins (C, NS3, NS5A) in hepatic oncogenesis. Hepatology Research.

[20] Lan K-H (2002). HCV NS5A interacts with p53 and inhibits p53-mediated apoptosis. Oncogene.

[21] Pawlotsky J, Germanidis G (1999). The non‐structural 5A protein of hepatitis C virus. Journal of viral hepatitis.

[22] Alessi DR (1997). Characterization of a 3-phosphoinositide-dependent protein kinase which phosphorylates and activates protein kinase Bα. Current biology.

[23] Altomare DA, Testa JR (2005). Perturbations of the AKT signaling pathway in human cancer. Oncogene.

[24] Cross DA (1994). The inhibition of glycogen synthase kinase-3 by insulin or insulin-like growth factor 1 in the rat skeletal muscle cell line L6 is blocked by wortmannin, but not by rapamycin: evidence that wortmannin blocks activation of the mitogen-activated protein kinase pathway in L6 cells between Ras and Raf. Biochemical Journal.

[25] Brazil DP, Yang Z-Z, Hemmings BA (2004). Advances in protein kinase B signalling: AKTion on multiple fronts. Trends in biochemical sciences.

[26] Plas DR, Thompson CB (2005). Akt-dependent transformation: there is more to growth than just surviving. Oncogene.

[27] Kondapaka SB, Zarnowski M, Yver DR, Sausville EA, Cushman SW (2004). 7-hydroxystaurosporine (UCN-01) inhibition of Akt Thr308 but not Ser473 phosphorylation: a basis for decreased insulin-stimulated glucose transport. Clinical cancer research.

[28] Pederson TM, Kramer DL, Rondinone CM (2001). Serine/threonine phosphorylation of IRS-1 triggers its degradation possible regulation by tyrosine phosphorylation. Diabetes.

